# Ultrasound-guided breast-sparing surgery to improve cosmetic outcomes and quality of life. A prospective multicentre randomised controlled clinical trial comparing ultrasound-guided surgery to traditional palpation-guided surgery (COBALT trial)

**DOI:** 10.1186/1471-2482-11-8

**Published:** 2011-03-16

**Authors:** Nicole MA Krekel, Barbara M Zonderhuis, Hermien WH Schreurs, Alexander MF Lopes Cardozo, Herman Rijna, Henk van der Veen, Sandra Muller, Pieter Poortman, Louise de Widt, Wilfred K de Roos, Anne Marie Bosch, Annette HM Taets van Amerongen, Elisabeth Bergers, Mecheline HM van der Linden, Elly SM de Lange de Klerk, Henri AH Winters, Sybren Meijer, Petrousjka MP van den Tol

**Affiliations:** 1 Department of Surgical Oncology, VU University Medical Centre, Amsterdam, the Netherlands; 2 Department of Surgical Oncology, Medical Centre Alkmaar, Alkmaar, the Netherlands; 3 Department of Surgical Oncology, Kennemer Gasthuis, Haarlem, the Netherlands; 4 Department of Surgical Oncology, Rode Kruis Hospital, Beverwijk, the Netherlands; 5 Department of Surgical Oncology, Waterland Hospital, Purmerend, the Netherlands; 6 Department of Surgical Oncology, Gelderse Vallei Hospital, Ede, the Netherlands; 7 Department of Radiology, VU University Medical Centre, Amsterdam, the Netherlands; 8 Department of Medical Psychology and Medical Oncology, VU University Medical Centre, Amsterdam, the Netherlands; 9 Department of Epidemiology and Biostatistics, VU University Medical Centre, Amsterdam, the Netherlands; 10 Department of Plastic and Reconstructive Surgery, VU University Medical Centre, Amsterdam, the Netherlands

## Abstract

**Background:**

Breast-conserving surgery for breast cancer was developed as a method to preserve healthy breast tissue, thereby improving cosmetic outcomes. Thus far, the primary aim of breast-conserving surgery has been the achievement of tumour-free resection margins and prevention of local recurrence, whereas the cosmetic outcome has been considered less important. Large studies have reported poor cosmetic outcomes in 20-40% of patients after breast-conserving surgery, with the volume of the resected breast tissue being the major determinant. There is clear evidence for the efficacy of ultrasonography in the resection of nonpalpable tumours. Surgical resection of palpable breast cancer is performed with guidance by intra-operative palpation. These palpation-guided excisions often result in an unnecessarily wide resection of adjacent healthy breast tissue, while the rate of tumour-involved resection margins is still high. It is hypothesised that the use of intra-operative ultrasonography in the excision of palpable breast cancer will improve the ability to spare healthy breast tissue while maintaining or even improving the oncological margin status. The aim of this study is to compare ultrasound-guided surgery for palpable tumours with the standard palpation-guided surgery in terms of the extent of healthy breast tissue resection, the percentage of tumour-free margins, cosmetic outcomes and quality of life.

**Methods/design:**

In this prospective multicentre randomised controlled clinical trial, 120 women who have been diagnosed with palpable early-stage (T1-2N0-1) primary invasive breast cancer and deemed suitable for breast-conserving surgery will be randomised between ultrasound-guided surgery and palpation-guided surgery. With this sample size, an expected 20% reduction of resected breast tissue and an 18% difference in tumour-free margins can be detected with a power of 80%. Secondary endpoints include cosmetic outcomes and quality of life. The rationale, study design and planned analyses are described.

**Conclusion:**

The COBALT trial is a prospective, multicentre, randomised controlled study to assess the efficacy of ultrasound-guided breast-conserving surgery in patients with palpable early-stage primary invasive breast cancer in terms of the sparing of breast tissue, oncological margin status, cosmetic outcomes and quality of life.

**Trial Registration Number:**

Netherlands Trial Register (NTR): NTR2579

## Background

Breast cancer is the most common cancer among women in the western world. It affects women of all ages and the lifetime risk of developing invasive breast cancer is 12-13%. The 5-year disease-free survival rate for node-negative breast cancer, however, is excellent (98%). Breast cancer surgery has changed dramatically over the past few decades. Breast-conserving therapy (BCT) was introduced in the 1970s and refers to the surgical removal of the breast tumour in all cases followed by radiotherapy to eradicate residual tumour cells. The introduction of the sentinel node procedure for nodal staging has avoided the morbidity of axillary lymph node dissection in the majority of patients. Several trials demonstrated comparable results with regard to disease-free and overall survival between mastectomy and BCT combined with radiotherapy, and the latter has become the standard of care for early-stage breast cancer. According to national guidelines, 75% of breast cancer patients are suitable for BCT. The main advantage of BCT over mastectomy is preservation of the breast with improved cosmetic outcomes [1-5].

Breast cancer and its treatment have many adverse side-effects, which may be both physical and psychological. Psychological distress is common in the breast cancer population and affects approximately 30% of patients. Fear of disease recurrence, concerns about future health and interruption of life plans often lead to anxiety and depression.

The cosmetic result is also a major determinant of psychological distress. Cosmetic outcome of the breast has a large impact on body image, and studies have shown that women with poor cosmetic outcomes as determined by pronounced breast asymmetry and skin alterations are impaired in their self-esteem, feelings of sexuality and quality of life. Furthermore, these women are more likely to feel stigmatised and have more symptoms of depression. Focusing on the best achievable cosmetic result will lead to a decrease in psychological distress [6-9].

Although BCT is considered the least invasive surgical method for treatment of breast cancer, cosmetic outcomes vary widely. Studies have reported satisfactory cosmetic results in only 60-80% of patients, with cosmetic failure rates up to 40%. Factors determining cosmetic outcome after BCT include the volume of resected breast tissue, the amount of radiotherapy, the site of the tumour in the breast, the type of incision and postoperative complications such as wound infection. Overall, a large volume of resected breast tissue is the major determinant of a poor cosmetic outcome. Regardless of the size of the breast, excision volumes exceeding 85 cm^3 ^result in a 50% rate of cosmetic failures, whereas smaller excision volumes result in only a 22% rate of cosmetic failures. Therefore, surgeons should excise the tumour with only a small volume of surrounding breast tissue [10-15].

As a practical and reasonable guideline during surgery, the aim is to achieve a safe and cosmetically acceptable resection margin of 5-10 mm. It should be noted that the size of the tumour-free resection margin (> 1 mm) is unrelated to local recurrence or overall survival. Higher risks of local recurrence have been shown only with evident involvement of the tumour on the inked resection margins. Therefore, there is no need to excise a tumour with a large volume of adjacent breast tissue. This is also stated by the Dutch national guidelines. Accurate excision leads to a smaller and more precise volume of surrounding breast tissue removal without compromising the minimal tumour-free margin [16-19].

**Figure 1:**
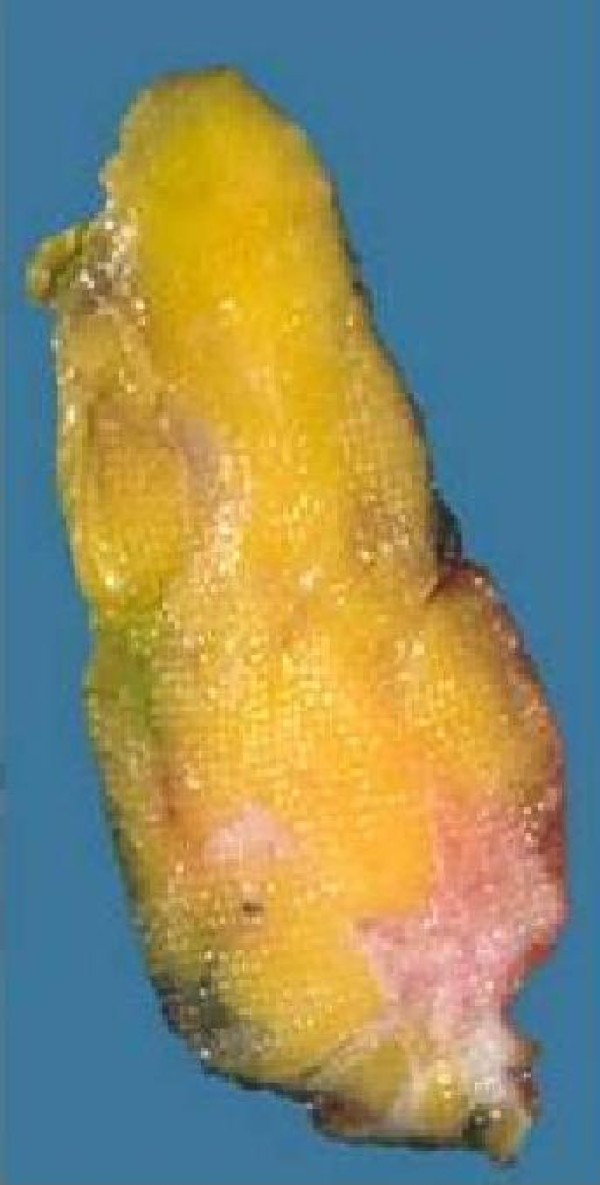
**Specimen with eccentric tumour localisation**.

In daily practice, the succes of the removal of palpable breast cancer is based on pre-operative imaging techniques and the experience of the surgeon. The surgeon is guided by intra-operative palpation without objective imaging during the surgery. The primary aim of the procedure is to achieve tumour-free resection margins and to prevent local recurrence; a secondary aim is a satisfactory cosmetic outcome. Avoiding inadequate resection margins and subsequent re-excision results in an unnecessarily wide resection of adjacent healthy breast tissue. Indeed, our recent large retrospective multicentre study demonstrated that during routine breast-conserving surgery, an excessive volume of breast tissue is excised in the majority of patients. The study participants (n = 726) underwent breast-conserving surgery in four hospitals in our region for invasive breast cancer in three consecutive years. The volumes of the excised specimens were calculated using histopathological reports. It was shown that in 33.6% (244/726) of the patients, the excised tissue volume exceeded 85 cm^3^, i.e., deteriorating the cosmetic outcome. In this group, 98.8% (241/244) had presented with an excision that exceeded the optimal resection volume (= the tumour volume plus a 1 cm margin of tumour-free breast tissue). In fact, in 54.9% (134/244) of these patients the tumour stage was T1 and a maximum resection volume of 33.51 cm^3 ^would have been sufficient (optimal resection volume for a tumour size of 2 cm and an added 1 cm margin of healthy breast tissue). Also, in most cases presenting with a tumour-free margin, the malignant lesion was located eccentrically in the excision volume, close to the nearest margin (Figure 1). Remarkably, the rate of focally positive or positive margins was higher for the palpable tumours than for the nonpalpable tumours (22.5% and 17.4%, respectively; P = 0.13) [20]. This might be caused by the lack of three-dimensional orientation with intra-operative visualisation in the excision of palpable tumours.

An easily available and feasible method to improve the amount of healthy breast tissue spared while ensuring tumour-free resection margins is intra-operative ultrasonography (US). US-guided surgery (USS) enables the surgeon to visualise the tumour during excision. Earlier studies have clearly shown the efficacy of USS for nonpalpable tumours. Rahusen et al. reported that USS, in which an experienced radiologist perfomed US, is superior to wire-guided surgery with respect to tumour-free resection margins (89% and 55% of cases, respectively). Snider et al. also showed an excellent rate of tumour-free resection margins using USS (82%) with a smaller volume of healthy breast tissue resection compared to wire-guided surgery (62.2 cm^3 ^and 81.1 cm^3^, respectively) [21-26]. Our retrospective multicentre study showed that USS for nonpalpable invasive breast cancer was more accurate than wire-localisation and Radio Occult Lesion Localisation (ROLL)-guided surgery because it optimised the surgeon's ability to obtain adequate margins (3.7%, 21.3%, and 25% tumour-involved margins, respectively, (P < 0.05). Intra-operative ultrasonography for nonpalpable tumours results in a dramatically lower rate of tumour-involved resection margins than palpation-guided surgery for palpable tumours (3.7% and 22.5%, respectively, (P < 0.05); unpublished data) [27]. To the best of our knowledge, only one study, by Moore et al., has evaluated the use of USS for treatment of palpable invasive ductal breast cancer. One group of patients received USS, and the other group underwent surgery without an imaging technique. The breast tissue spared and margin status were both improved with US [28].

In general, all palpable breast masses are visible by US. Given that the efficacy of USS has already been shown for nonpalpable tumours, we hypothesise that USS will also be useful for palpable tumours with regard to improving the amount of healthy breast tissue spared, cosmetic outcomes and quality of life while still maintaining or even increasing tumour-free resection margins.

## Methods/Design

### Design

This study will be a multicentre prospective randomised clinical trial. Eligible patients will be randomised for either ultrasound-guided surgery (USS) or palpation-guided surgery (PGS).

### Subjects and patient selection

One hundred and twenty patients will be recruited over a period of six months at a university medical center and several medium to large hospitals in the Netherlands. All women aged 25-75 yrs who are diagnosed with palpable early-stage (T1-2N0-1) invasive breast cancer in the trial centres will be invited to participate in this study. Breast cancer will be diagnosed with physical examination, mammography (2R) and US, and occasionally MRI of the breast and axilla. The diagnosis of invasive (ductal or lobular) breast cancer will be established with image-guided core needle biopsy or cytological puncture. All patients will be discussed in a multidisciplinary team, and suitable for BCT according to national guidelines. Participants will not have a history of prior mammary surgery of the affected breast, radiation therapy or neo-adjuvant therapy. Participants will have ASA Classification I-III and will be well-informed having signed an informed consent form.

Full ethical approval is obtained for this study from the Investigational Review Board of the VU University Medical Centre, including the Medical Ethical Review Board.

### Study procedures

#### Pre-operative

Prior to surgery, patients will be informed about this trial by both written and oral explanations. Informed consent will be obtained. Subsequently, the patients will complete the quality of life questionnaire, EORTC QLQ-C30/-BR23. The principal investigator will randomly assign the participants to either of the two treatment modalities by using a digital randomisation program. Group I will undergo PGS and group II USS. Stratification by institute will be performed to ensure balanced allocation between the two treatment modalities in each institution.

#### Surgery

Surgery will be performed under general anaesthesia by dedicated oncological breast surgeons or by surgical residents under their close supervision. The surgery will start with the axillary procedure. In clinically node-negative patients surgery of the axilla consists of a single sentinel node procedure. For the sentinel node procedure, a triple method will be used, consisting of combined lymphoscintigraphy (Tc99m colloidal albumin [Nanocoll^®^]), Patent Blue V^® ^(Guerbet, Aulnay-Sous Bois, France) dye injection, and gamma probe detection[2]. In some institutions, the sentinel node is sent for frozen section study, and if metastases are diagnosed, axillary lymph node dissection is performed during the same procedure. Node-positive patients who are pre-operatively confirmed by US-guided cytological puncture will undergo an axillary lymph node dissection. Axillary lymph node dissection is a routine procedure, consisting of a classic level I and II dissection. After the axillary procedure, surgery of the breast is performed. The aim in both the USS-group and the PGS-group will be to obtain a rim of 1 cm of healthy adjacent breast tissue around the malignant breast lesion.

#### Palpation-guided surgery

In the PGS group, tumour excision will be guided by the palpation of the surgeon in the standard manner. The index finger will be used to palpate the mass, retract it and guide the dissection. In this procedure, the adequacy of the resection is based on the experience of the surgeon without objective imaging during surgery.

#### Ultrasound-guided surgery

**Figure 2:**
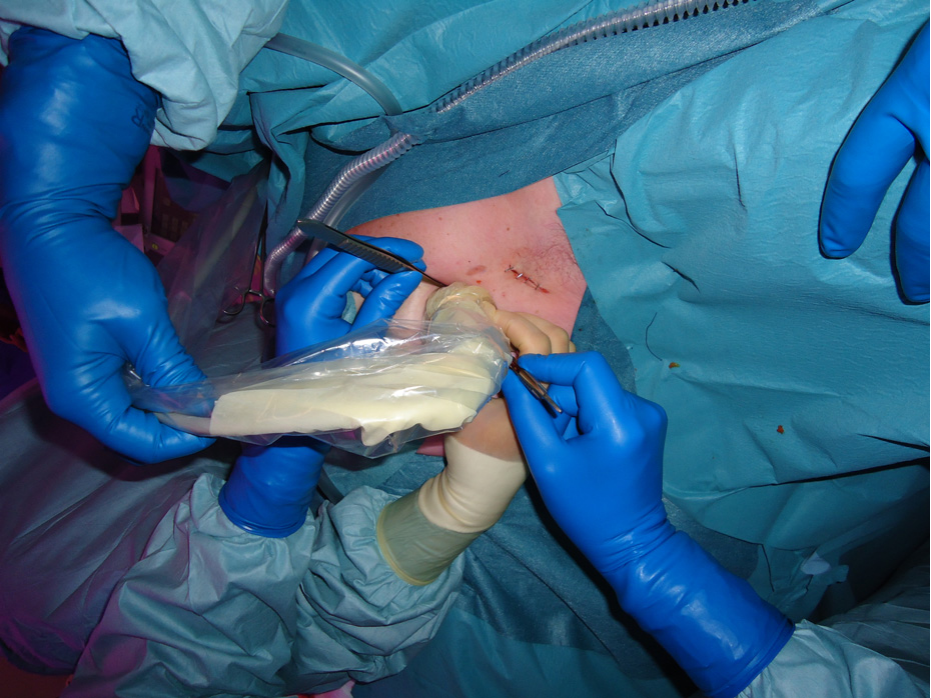
**Tumour localising by ultrasound imaging**.

**Figure 3:**
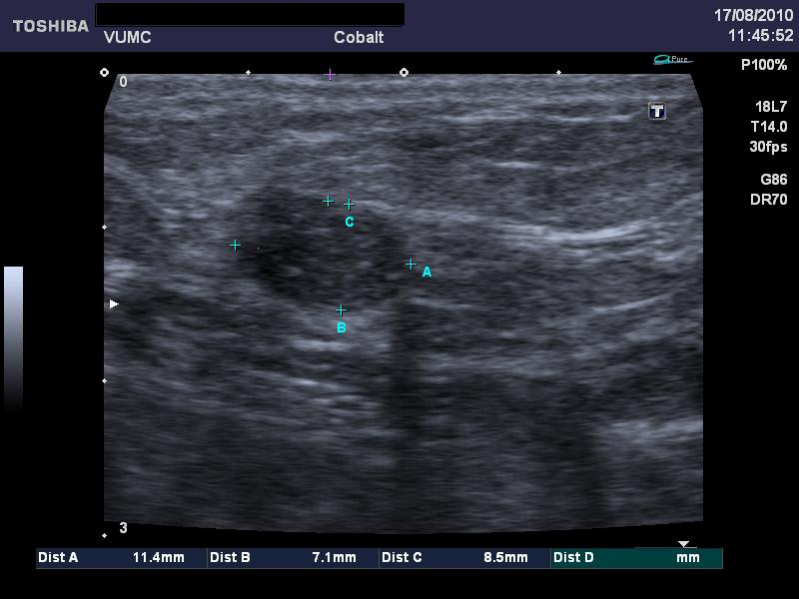
**Ultrasound image on screen**.

**Figure 4:**
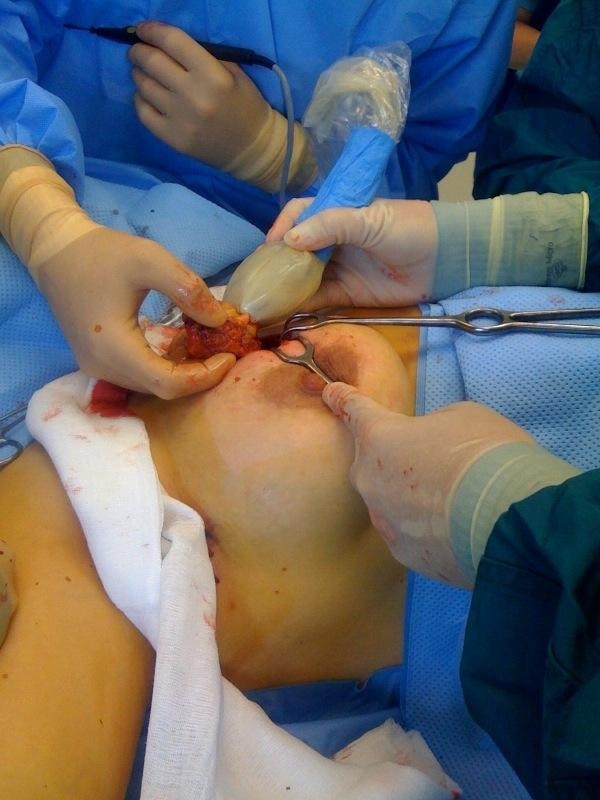
**The ultrasound is applied repeatedly in the wound**.

**Figure 5:**
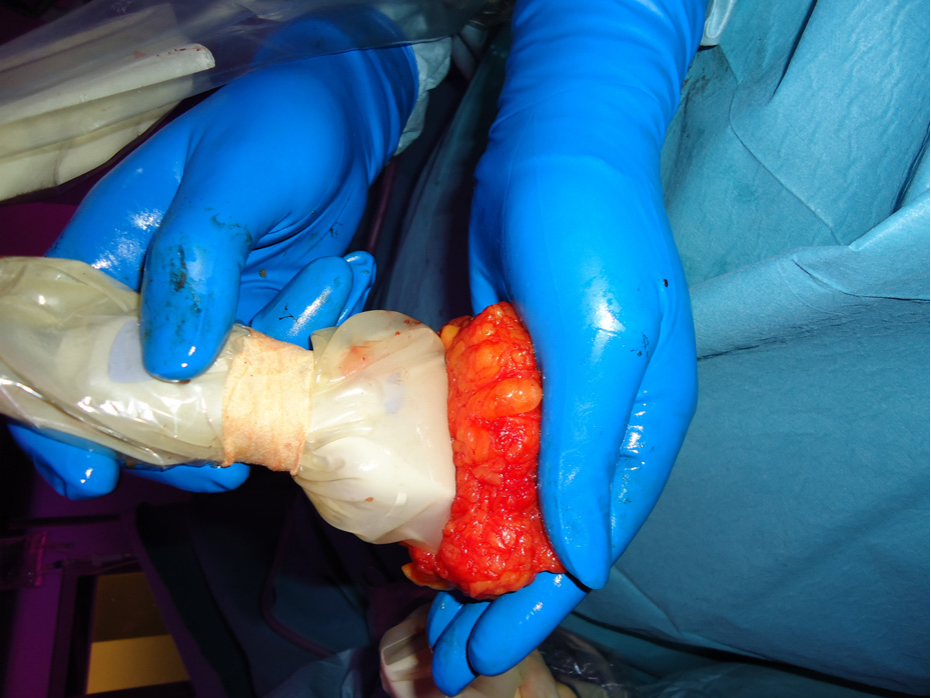
**After excision, completeness of the specimen is checked by ultrasonography**.

In the USS group, tumour excision will be performed by the surgeon with US guidance in collaboration with an experienced radiologist. Prior to surgery, the surgeon will carry out an US of the lesion under direct supervision of the radiologist. During the surgery, the radiologist will be present either in the operating theatre or in the radiology department. The surgical procedure will start with the standard sentinel node procedure, after which the US-guided lumpectomy will be performed. USS will be performed using a THI 14-MHz US probe (Toshiba Viamo portable ultrasound system, Japan). The probe is coupled to a mobile US unit and covered with a sterile sheath that enables it to be used in the surgical wound. The lesion will be carefully localised in the breast by palpation and US imaging (Figure 2). The localisation of the lesion will be compared to the pre-operative images. The breast tissue will be positioned in such a way that the lesion is located as close to the skin surface as possible, and the breast will be fixed in that position throughout the procedure by hand or using tape. The tumour size, lesion-to-skin distance and lesion-to-fascia distance will be measured in millimetres by US. The localisation of the tumour will be precisely marked on the skin, and the incision will be made. After the incision, the skin over the lesion will be dissected from the subcutaneous tissue, and the US probe will be positioned in the wound to reassess the position of the lesion. Also, the index finger will be used to palpate the mass, retract it and guide the dissection. Dissection is continued posteriorly in the plane between the breast and the pectoral fascia. To achieve adequate margins, US will be applied repeatedly in the wound from different angles while continuously monitoring the location and depth of the tumour (Figure 3, 4). Subsequently, a spherical lump of breast tissue will be excised. It is recommended to place a "pool suture" around the tumour under US guidance to facilitate the excision. After completion of the excision, the specimen will be scanned ex vivo by US to assess the completeness of the excision (Figure 4, 5).

The orientation of the specimen will be preserved with marking sutures such that positive resection margins can be identified and re-excised if necessary. Haemostasis will be obtained, and drains will not be used. If requested, titanium clips will be placed in each quadrant to guide radiotherapy. Subcutaneous tissue will be closed with dissolvable stitches, and the skin will be closed with stitches or staples.

### Outcome parameters (Table 1)

**Table 1:** Outcome Parameters

Patient	Age
	Race
	Breast size
	Medical history
**Pre-operative**	Experience of the surgeon
	Complications
	Duration of the procedure
	Cost of the procedure
	Type of surgery in the axilla (SN or ALND)
	Adequate margins based on US ex vivo
	Specimen volume (fluid displacement

**Histological**	Surgical specimen volume
	Tumour volume (diameter) within the specimen
	Margin status
	Shortest distance to the nearest resection margin

**Adjuvant therapy**	Radiotherapy (dose, boost)
	Chemotherapy
	Hormonal therapy

**Two weeks post-operatively**	(Wound) complications

**Three and six months post-operatively**	Cosmetic outcome measurements
	Quality of life (questionnaires)

#### Perioperative parameters

The experience of the surgical residents, complications, the duration of procedure (i.e. time from the incision to wound closure; time needed for the axillary surgery is excluded) and costs of the procedure are registered.

After excision the volume of the specimen will be measured in the operating room by fluid displacement. The specimen will be submerged in an Erlenmeyer flask filled with normal saline at 37^176;^C. The volume of fluid displaced equals the volume of the specimen. If re-excision is performed during the surgery, the volume of the reexcised specimen will be calculated separately and subsequently added to the originally excised specimen.

#### Pathology

This study would not influence current pathological examination.

After the placement of wire markers on the fresh specimen, the specimen is sent together with the sentinel node immediately to the pathologist. The pathological examination of the surgical specimen and sentinel node is as follows:

The three dimensions of the surgical specimen are measured macroscopically. Specimens will be carefully inked and cut in 4-mm slices. Subsequently, the tumour diameter, the site of the tumour in the surgical specimen, the margin status and the smallest distance to the tumour-free resection margin will be measured (mm). The margin status and the smallest distance to the tumour-free resection margin will be measured more precisely using microscopy (mm), and finally, tumour characteristics (e.g. tumour differentiation, receptor status) will be recorded. The three dimensions of the sentinel node are measured macroscopically. Lymph nodes measuring < 5 mm are examined in toto, nodes measuring > 5 mm are cut. Microscopically the presence and size of metastasis are defined (mm).

### Calculations

The ratio of the resected volume to the optimal resected volume can be calculated from the diameter of the tumour and the three dimensions of the specimen.

Three assumptions are made:

1) the tumour is spherical (using the radius (= ½ diameter),

2) in an optimal resection volume, a margin of 1 cm is excised around the tumour and

3) the excised specimen is ellipsoid.

The volume of the excised specimen will be measured during surgery by fluid displacement and the histopathological ellipsoid calculation will serve as a control.

**Figure 6:**
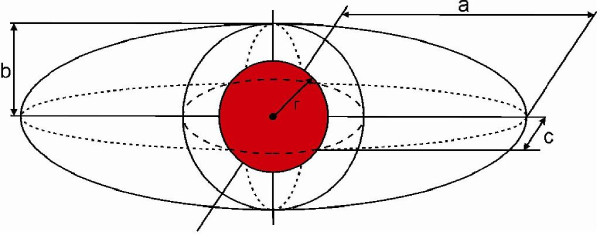
**Comparing the specimen volume with the optimal excision volume**.

- The volume of the tumour will be calculated by 4/3πr ^3^.

- The optimal volume required for excision will be calculated by adding a resection margin of 1 cm to the lesion radius and converting this value into a spherical volume using the formula 4/3π(r + 1 cm) ^3^.

- The volume of the surgical specimen will be calculated using the formula 4/3πa b c (with a, b and c as given in the pathology report). The specimen volume will be compared with the optimal excision volume, resulting in a ratio (Figure 6) [29].

#### Postoperative

This study would not influence current postoperative treatment.

The current postoperative treatment is as follows:

Within two weeks after surgery, patients visit the outpatient clinic. Pathological results and further treatment (with multidisciplinary approach) are explained in detail. Complications are registered and treated, with special attention given to wound healing.

Approximately six weeks after surgery, all patients receive radiotherapy of the affected breast. Patients younger than 50 years old are treated with whole-breast irradiation (50 Gy in 25 fractions) including a simultaneously integrated boost (SIB), for a total dose of 68,75 Gy. Patients over 50 years old receive 40 Gy in 15 fractions with a SIB, for a total dose of 50,25 Gy. Radiotherapy strategies may vary slightly according to institutional guidelines.

If necessary, a medical oncologist is consulted for the administration of systemic therapy. Adjuvant therapy is administered according to national guidelines. When multiple axillary lymph node metastases are diagnosed, patients will receive adjuvant therapy prior to radiotherapy.

#### Cosmetic Analysis

**Figure 7:**
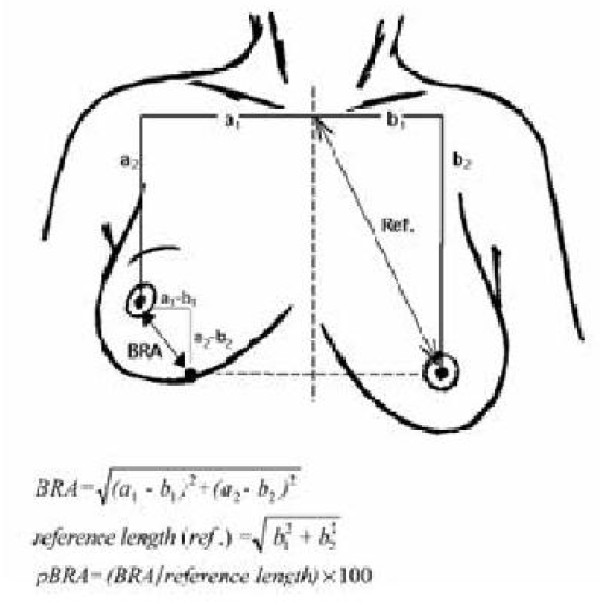
**Breast Retraction Assessment**.

During the follow-up visits three and six months after surgery, the cosmetic outcome will be evaluated. Standard 4-point view digital photographs, including the suprasternal notch, will be taken. These photographs will be scored by a panel consisting of six persons (including both professionals and non-professionals). The items scored will be the overall result, the appearance of the surgical scar, breast size, breast shape, nipple position and the shape of areola. The treated breast will be compared to the untreated breast, using the 4-point Lickert scale. Objective cosmetic analysis will be performed with the Breast Retraction Assessment (BRA) or comparable objective software. BRA values are calculated using measurements from the frontal view photographs and quantity the amount of retraction of the treated breast compared to the healthy contralateral breast by measuring nipple displacement. A large BRA value corresponds to increased asymmetry between the breasts and to poor cosmetic results (Figure 7). Subsequently, the patients' opinions concerning cosmetic outcome will be assessed using patient self-evaluation. Long-term follow-up (3 yrs) will probably be studied in a future research project.

#### Quality of Life questionnaire

The EORTC QLQ-C30/-BR23 questionnaire will be completed by the patient prior to surgery and during follow-up visits three and six months after surgery. Using a personalised interview three and six months after surgery, patients' feelings concerning the cosmetic results of their breasts will be assessed more specifically. Results will be analysed over time.

### Follow-up

Oncological follow-up will continue according to national guidelines (every three months for the first two years). One year after surgery, follow-up will include no study issues. If the results of this study are promising, long-term results will be analysed (3 yrs of follow-up).

### Sample size calculation

The sample size was calculated based on the primary endpoint: volume of resected breast tissue. Previous studies have shown that excision volumes exceeding 85 cm^3 ^result in significantly higher cosmetic failure rates. In our recent retrospective multicentre study it was found that in 33.9% (244/726) of the patients the excision volumes exceeded 85 cm^3^. In 98.8% (241/244) of these patients, the CRR was > 1.0, implying that excess tissue was excised [20]. It can therefore be concluded that, in 33.2% (241/726) of all patients, excessive tissue resection resulted in impairment of the cosmetic outcome. Assuming that this excessive resection can be avoided with US-guided surgery, it is expected that only 10% of women will have excessive breast tissue resection (a ratio of > 1.0) that results in a worse cosmetic outcome (resection volume > 85 cm^3^). Therefore, we assume a difference in resected volume of 22.3% in favour of the US group. With a statistical power of 80% to detect this 22.3% improvement as significant (P < 0.05), we will require 57 patients in the palpation group and 57 patients in the US group. The margin status is currently involved in 22.5% of palpable breast tumour excisions. To improve this rate to 5%, we will require 60 patients in the palpation group and 60 patients in the US group. Therefore, the target sample size will be 120 patients.

### Timeline

The inclusion period will run from October 2010 to July 2011. The EORTC QLQ-C30/-BR23 questionnaire will be completed by the patient prior to surgery. Surgery will be scheduled 1-2 weeks after diagnosis. During a follow-up visit to the outpatient clinic, usually 1-2 weeks after discharge, complications will be recorded and treated. Three and six months after surgery, information will be collected from each patient concerning the cosmetic outcome and quality of life, and photographs will be taken. Patients can withdraw from the study at any time during the study period.

### Statistical analysis

Descriptive statistical methods will be applied. Statistical software (i.e. SPSS 15.0.) will be used for analysis. The 95% confidence intervals of the differences between the two groups will be calculated. The Chi-square test will be used for comparisons of dichotomous variables. The Students' t-test will be used, where appropriate, for comparisons of continuous variables. The Fisher's exact test will be performed where applicable. Multiple regression analysis will be used for comparisons of independent variables. The patient, tumour and treatment related differences will be calculated for the two treatment strategies.

An interim analysis will be performed after the inclusion of 60 patients (30 patients in each group). The final data analysis will be performed after the surgeries of the total of all 120 patients are completed. Definitive results are expected in December 2011.

## Discussion and Conclusion

The goals of breast-conserving surgery are to obtain adequate margins and good cosmetic results. Both goals are poorly defined, and there is no universal acceptable standard. According to Dutch national guidelines, a negative margin is defined as a margin of ≥ 1 mm of normal tissue intervening between the tumour and the edge of the specimen. Resections are described as focally involved when cancer cells invade the resection margin in a maximum of two microscopic slides or when margins include < 1 mm of normal tissue. The margins are described as involved or positive when microscopic cancer cells are situated on the inked resection border. It has been well-established that the status of the margin affects the local recurrence rate. Therefore, re-excision is indicated for involved margins. Vrieling et al. showed that patients with focally involved margins have lower rates of recurrence when treated with a higher radiation boost dose, equal to patients with adequate margins. Therefore, a higher boost dose is given to patients with focally involved margins without further surgery. This has been confirmed by national guidelines [15-19].

To analyse cosmetic outcomes, frequently used subjective methods are patient self-evaluation and panel evaluation. Patient self-evaluation is a valuable method as the patient's opinion is of great importance; however, patients tend to report consistently better scores than professionals. Panel-evaluation, consisting of a panel of six professionals and non-professionals, has proven to be the most reliable subjective method. The panel evaluates 4-point view pictures of the breasts [8,13]. Breast Retraction Assessment (BRA) is a well-validated and frequently used objective method to evaluate breast asymmetry. The BRA is reliable and minimally time-consuming; however, it is only correlated moderately for tumours located in the lower quadrant, and skin changes (e.g. disturbing scars or telangiectasias) are not taken into account. In the framework of the EORTC trial, Vrieling et al. compared these different methods of cosmetic outcome assessment. The BRA is recommended for comparing the cosmetic outcomes between two different approaches to BCT and for analysing cosmetic changes over time. The panel evaluation gives the best measure of the overall cosmetic result. Therefore, in order to assess cosmetic outcomes, the most appropriate method is to combine the panel evaluation and the BRA into an overall score. Patient self-evaluation is necessary with regard to quality of life [30,31].

The excision of nonpalpable breast cancer can be performed with guidance from several tools. The wire-localisation (WL) is still the gold standard. The WL procedure is technically demanding and depends on both the wire placement by the radiologist and on the experience and three-dimensional orientation of the surgeon. The insertion of the wire can be uncomfortable for the patient; Furthermore, there is a risk of wire migration between the time of insertion and the beginning of the surgery. Other less frequently used techniques are emerging, including the use of radioactive seed implants, an electrosurgical loop device and Radio-guided Occult Lesion Localisation (ROLL), which uses a radioactive pharmaceutical that is injected into the tumour pre-operatively. A gamma probe is used to guide the surgical resection. A drawback is that these guidance tools are invasive. Currently, these techniques are being validated [32-34]. Intra-operative ultrasonography (US) was introduced in 1988 as an easily available and patient-comfortable method of excising a tumour under direct vision. Using intra-operative US, surgeons can localise and guide the excision of non-palpable lesions, without the need for additional interventions before surgery. After specimen removal, the US is valuable to confirm excision and check margin clearance before wound closure. However, a possible restriction is the arrangement of a radiologist's presence in the operating theatre. In our multicentre study wire-localisation (WL), ROLL and USS were retrospectively compared. USS was clearly the most effective method for the excision of nonpalpable tumours [27]. Moreover, a number of studies such as Rahusen et al. and Snider et al. have clearly demonstrated that intra-operative US guidance has considerable advantages over wire-guided excision, including reduced pre-operative stress and discomfort for patients and decreased operating room time. Most importantly, intra-operative US guidance resulted in improved resection margins and smaller excision volumes.

High-frequency US shows most lesions, and in general, all palpable breast lesions are visible with ultrasonography (US). Therefore, intra-operative US is applicable in the majority of women with a palpable breast cancer [21-26,28,33].

Intra-operative ultrasonography will contribute to improved cosmetic outcomes by:

1. Smaller volumes of resected breast tissue

2. Improvement of margin status

- avoiding a higher boost dose for focally involved margins

- avoiding re-excision or even mastectomy for involved margins

Approximately 9000 out of 13000 breast cancer patients are diagnosed with a palpable breast cancer in the Netherlands each year, and around 5000 patients undergo breast-conserving surgery for a palpable breast cancer. An improvement of cosmetic outcomes in an estimated n = 1115 (22.3%) of all patients treated with breast-conserving surgery can be achieved by USS. Also, an estimated n = 875 (17.5%) less operations will be necessary after treatment by US-guided surgery (A re-operation costs about € 7 000, -, so eventually € 6 125 000, - might be saved by this method each year).

In conclusion, this randomised controlled trial aims to demonstrate the superiority of USS versus PGS for the treatment of patients with palpable breast cancer in terms of the sparing of breast tissue, oncological margin status, cosmetic outcomes and quality of life.

## Abbreviations

BCT: Breast-conserving therapy; US: Ultrasonography or ultrasound; WL: Wire localisation; ROLL: Radio-guided Occult Lesion Localisation; USS: Ultrasound-guided surgery; PGS: Palpation-guided surgery; METc: Medical Research Ethics Committee (MREC) (in Dutch: Medisch Ethische Toetsingscommissie (METc))

## Competing interests

The authors declare that they have no competing interests.

## Authors' contributions

NMAK, BMZ, SM, SM, and PMPT drafted the manuscript and made substantial contributions to the acquisition of funding. NMAK, BMZ, SM, HWHS, HR, HV, SM, and PMPT participated in the collection of all retrospective data. NMAK, BMZ, SM, HWHS, AMFLC, WKR, AMB, HR, PP, LW, HV, HAHW, SM, and PMPT have made substantial contribution to the conception and design of the study. SM, HWHS, AMFLC, WKR, AMB, HR, PP, LW, HV, SM, and PMPT have made great efforts to be trained in the ultra-sound guided breast surgery techniques, since they are the participating surgeons. They are also crucial in the inclusion of patients and the collection of all data of the randomised controlled trial. AHMTA and EB made contribution to the conception of the study and the design of the study, in particular for the technical part of ultrasonography. They teach the surgeons how to use the ultrasound. ML participated in the design of the quality of life assessment, constructed the questionnaires, and will carry out all quality of life interviews. ESMLK participated in the design of the study and carried out the statistical analysis, the interpretation of data and sample size calculations. HAHW participated in the design of the cosmetic assessment, and will conduct the panel evaluation. All authors read and approved the submitted version of the manuscript. SM, and PMPT have given final approval of the final version to be published.

## Pre-publication history

The pre-publication history for this paper can be accessed here:

http://www.biomedcentral.com/1471-2482/11/8/prepub
